# Metabolic Syndrome as a Determinant of Bidirectional Transitions Between Frailty States: Evidence From the Whitehall II Study

**DOI:** 10.1002/jcsm.70341

**Published:** 2026-07-08

**Authors:** Steve Kevin Njouonkep Sime, Bi Boli Anselme Stéphane Irié, Etienne Jules, Léopold Fezeu K., Estelle Pujos‐Guillot, Blandine Comte

**Affiliations:** ^1^ University Clermont Auvergne, INRAE, UNH, Platform of Metabolism Exploration, MetaboHUB Clermont Saint‐Genès Champanelle France; ^2^ Sorbonne Paris Cité Epidemiology and Statistics Research Center (CRESS), Nutritional Epidemiology Research Team (EREN), Inserm U1153, INRAE U1125, Cnam, University of Paris Bobigny France

**Keywords:** aging, bidirectional transitions, frailty, metabolic syndrome, multi‐state Markov model, older adults

## Abstract

**Background:**

Understanding interactions between geriatric syndromes is central for promoting healthy aging. Frailty is among the most relevant, as it predicts disability, falls, hospitalization and mortality. In addition, emerging evidence also indicates that metabolic factors play a key role in the frailty development. Among them, metabolic syndrome (MetS) has been examined because of shared metabolic, inflammatory and endocrine mechanisms. Previous studies reported that MetS and its components increase the risk of pre‐frailty and frailty. However, the role of MetS in the dynamics of frailty trajectories, namely, transitions between frailty states, remains poorly understood. Using data from the Whitehall II cohort, we examined the influence of MetS and its components on bidirectional transitions between the frailty states.

**Methods:**

Data from Phases 9, 11 and 12 of the Whitehall II cohort (*n* = 10 308) were analysed. MetS and its five components were defined at baseline according to the consensus definition. Frailty states were classified using Fried's phenotype: robust (0 criteria), pre‐frailty 1 (PF‐1; 1 criterion), pre‐frailty 2 (PF‐2; 2 criteria) and frailty (≥ 3 criteria). A multi‐state Markov model with death as an absorbing competing state was used to estimate transition intensities and probabilities. Transition‐specific hazard ratio (HR) for MetS and its components were adjusted for sociodemographic, lifestyle and health‐related factors.

**Results:**

A total of 4750 participants (mean age: 64.6 years; 73.9% men) were followed for an average of 6.9 years. At baseline, 58.8% were robust, 29.4% were PF‐1, 9.2% were PF‐2, and 2.5% were frail; 36.9% had MetS. Recovery transitions were more frequent than deteriorations: Among PF‐1 individuals, recovery to robustness occurred at an intensity of 0.20 (95% CI: 0.18–0.22) versus 0.15 (0.14–0.17) for progression to PF‐2. In PF‐2, recovery to PF‐1 was 0.30 (0.26–0.35), versus 0.15 (0.12–0.18) for progression to frailty. Overall, MetS was not significantly associated with transitions. However, abdominal obesity increased the hazard of transitioning from robustness to PF‐1 by 27% (HR = 1.27; 1.09–1.48; *p* = 0.001) and reduced recovery from PF‐2 to PF‐1 by 28% (HR = 0.72; 0.52–0.99; *p* = 0.04) and from frailty to PF‐2 by 50% (HR = 0.50; 0.29–0.87; *p* = 0.014). Hyperglycemia reduced recovery from PF‐1 to robustness by 28% (HR: 0.72; 0.59–0.88; *p* < 0.001). Other components, including hypertension, high blood levels of triglycerides and low blood levels of HDL‐cholesterol, showed no significant associations.

**Conclusion:**

MetS overall was not significantly associated with frailty transitions. Nevertheless, abdominal obesity and hyperglycemia were linked to impaired recovery and accelerated progression toward frailty. Early intervention targeting these metabolic disturbances may support healthier aging trajectories.

## 1 Introduction

The global demographic shift toward an aging population poses major challenges for healthcare systems, driven by the increasing prevalence of age‐related vulnerability syndromes [1]. Among these, frailty is one of the most critical, reflecting a decline in physiological reserves and an increased state of vulnerability associated with aging [2]. Frailty is a strong predictor of adverse health outcomes, including dependency, falls, loss of autonomy, hospitalization and mortality [3]. In community‐dwelling older adults worldwide, frailty affects up to 20% of individuals, whereas pre‐frailty, a preliminary state, may affect as many as 50% [4]. Identifying modifiable determinants along the continuum from robustness to dependency has therefore become a major public health priority [5].

Today, there is good evidence that changes in metabolism play an important role in aging, in particular due to environmental factors, such as lifestyles and nutrition [6]. Given these close links, there is a growing interest in studying the relationship between metabolic diseases and frailty in aging. In particular, among the biological factors contributing to frailty, metabolic syndrome (MetS) represents a major age‐related condition characterized by a cluster of metabolic disturbances, including abdominal obesity, elevated blood pressure, dyslipidaemia (high blood triglycerides and/or low blood HDL‐cholesterol) and hyperglycaemia [7, 8]. MetS is a well‐established driver of type 2 diabetes, cardiovascular diseases and premature mortality [7, 8]. Beyond these impacts, MetS has also been implicated in frailty development, as both share overlapping pathophysiological mechanisms, such as chronic low‐grade inflammation, insulin resistance and oxidative stress, that contribute to impaired homeostasis and loss of physiological resilience [9, 10, 11, 12, 13].

In addition to their frequent co‐occurrence in advanced age, increasing evidence from both cross‐sectional and longitudinal studies indicates that MetS and its components are associated with a higher risk of developing pre‐frailty and frailty [9, 10, 11, 12, 13, 14, 15]. This relationship has been reinforced by large‐scale genetic studies, including Mendelian randomization analyses involving more than half a million participants [16]. Despite this expanding body of evidence, little is known about how MetS influences the dynamics of frailty trajectories, specifically, the bidirectional transitions between robustness, pre‐frailty and frailty. Understanding these bidirectional transitions is crucial not only for predicting the progression of frailty but also for identifying recovery factors, thereby informing interventions that may prevent frailty or even reverse frailty [17]. The present study was designed to address this gap by investigating the influence of MetS and its individual components on bidirectional transitions across frailty states over time. Using longitudinal data from the Whitehall II cohort, this study aimed to generate novel insights into the metabolic determinants of frailty dynamics, with potential implications for more effective prevention and management of age‐related vulnerability.

## 2 Materials and Methods

### 2.1 Study Population

This study used data from the Whitehall II cohort (https://www.ucl.ac.uk/psychiatry/research/mental‐health‐older‐people/whitehall‐ii), a prospective longitudinal study initiated in 1985 to investigate social determinants of health, particularly social inequalities among British civil servants. The original cohort comprised 10 308 participants (6895 men and 3413 women), aged 35–55 years at recruitment, drawn from 20 London‐based civil service departments. To date, 13 phases of data collection have been completed. Participants underwent clinical examinations and completed self‐administered questionnaires covering sociodemographic characteristics, health status and lifestyle. Data collection alternated between questionnaires‐only phases and phases combining questionnaires with clinical assessments. The study is coordinated by University College London (UCL) and received ethical approval from the UCL Research Ethics Committee for all phases. Written informed consent was obtained from all participants.

**FIGURE 1:**
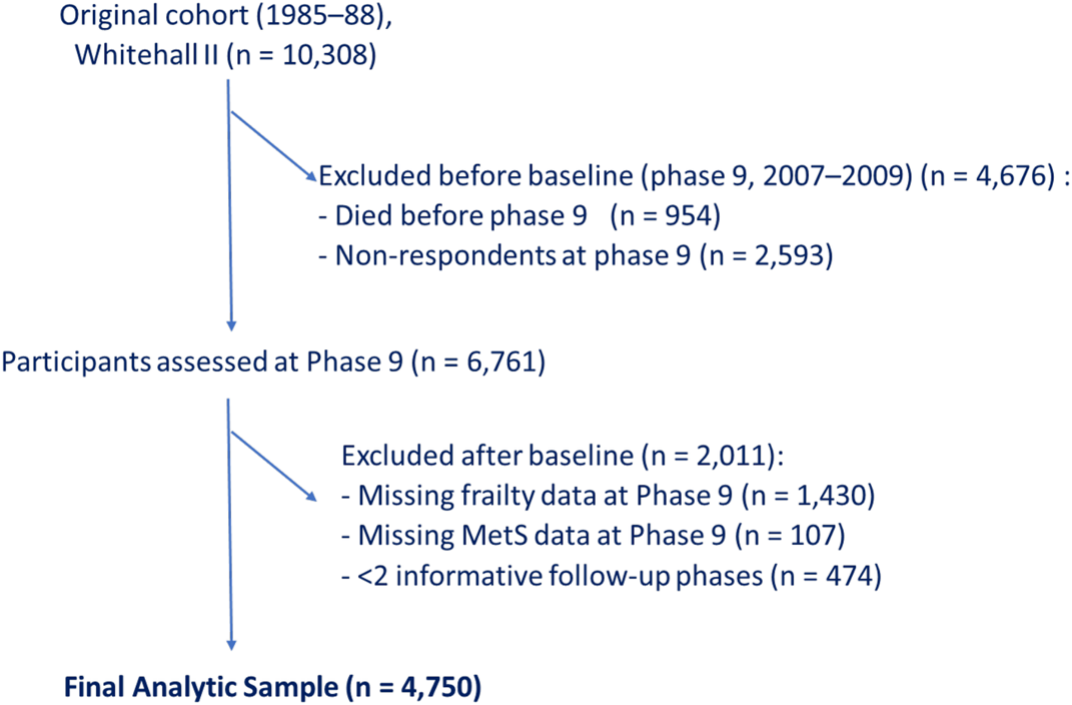
Flow chart of participants included in the analysis. Phase 9 of the Whitehall II study (2007–2009) was considered the baseline. Participants who were dead, were non‐respondents at this phase, or who lacked frailty, metabolic syndrome data, sufficient follow‐up information were excluded.

For the present analysis, Phase 9 (2007–2009) served as the baseline, as it is the first phase including all the components required to construct a complete and harmonized Fried frailty phenotype that were available and validated [18]. Phase 9 also corresponds to the period when all participants have reached an age range of 57–77 years, in which frailty and prefrailty become sufficiently prevalent to allow meaningful modelling of transitions [4]. Of the original 10 308 participants, 954 were dead and 2593 were non‐respondents prior to this phase, leaving 6761 participants eligible at baseline. Data from participants followed through Phases 11 (2012–2013) and 12 (2015–2016) were used to capture frailty transitions. Eligibility criteria included valid frailty data at baseline and at least one additional informative phase, defined as either having valid frailty data or being deceased at follow‐up, thereby allowing observation of a transition. Participants were excluded if they had frailty and MetS missing data at baseline (*n* = 1430 and 107, respectively), or contributed to fewer than two informative phases overall (*n* = 474). The final analytic sample consisted of 4750 participants (Figure 1).

### 2.2 Exposure

MetS was the primary exposure, assessed at baseline (Phase 9, 2007–2009) according to the consensus threshold definition [7]. MetS was defined as the presence of at least three of the following components: (1) elevated fasting blood glucose (≥ 5.6 mM or use of glucose‐lowering medication); (2) low blood HDL‐cholesterol (< 1.0 mM for men, < 1.3 mM for women or use of lipid‐modifying medication); (3) elevated blood pressure (systolic ≥ 130 mmHg and/or diastolic ≥ 85 mmHg, or use of antihypertensive medication); (4) high blood triglycerides (≥ 1.7 mM, or use of lipid‐modifying medication); (5) abdominal obesity (waist circumference ≥ 102 cm for men, ≥ 88 cm for women). The main analysis examined MetS as a predictor of frailty‐states transitions over follow‐up. In additional analyses, each MetS component was analysed separately to assess its specific association with frailty transitions.

### 2.3 Outcome

The events of interest were transitions between frailty‐related states: robust, pre‐frailty 1 (PF‐1; one positive criterion), pre‐frailty 2 (PF‐2; two positive criteria), frailty (three or more positive criteria) and death. Death was considered an absorbing state to account for competing risk. Frailty was defined using Fried's phenotype [2], with pre‐frailty further subdivided into PF‐1 and PF‐2. The five criteria were assessed as follows:

1. **Grip strength:** measured using a Smedley hand grip dynamometer. BMI‐ and sex‐specific cut‐offs defined low grip strength as ≤ 29 kg (BMI ≤ 24 kg/m^2^), ≤ 30 kg (BMI 24.1–28), ≤ 32 kg (BMI > 28) for men, and ≤ 17 kg (BMI ≤ 23), ≤ 17.3 kg (BMI 23.1–26), ≤ 18 kg (BMI 26.1–29), ≤ 21 kg (BMI > 29) for women.
2. **Walking speed:** Assessed using established thresholds. Participants were classified as having a slow walking speed if the time to walk 2.4 m was ≥ 3.73 s (for men ≤ 173 cm or women ≤ 159 cm) or ≥ 3.20 s (for men > 173 cm or women > 159 cm).
3. **Physical activity:** Low levels of physical activity were defined as an energy expenditure of < 383 kcal/week for men and < 270 kcal/week for women, calculated within the cohort as previously described [19].
4. **Exhaustion:** assessed using two items from the Centre for Epidemiologic Studies—Depression (CES‐D) scale [20]: ‘I felt that everything I did was an effort’ and ‘I could not get going’. Participants who answered ‘occasionally or moderate amount of the time (3‐4 days)’ or ‘most or all of the time (5‐7 days)’ to either of the items were categorized as exhausted.
5. **Weight loss:** originally defined as an unintentional loss of ≥ 5% of body weight over the past year. In the absence of such data, we used a proxy definition: a ≥ 10% decrease compared with the weight measured at the previous study phase, an adaptation used and validated in previous Whitehall II publications [18] and in other cohort studies, including work by Fried and colleagues in the Women's Health Aging Study I [21].

### 2.4 Covariates

Covariates were baseline characteristics selected based on a comprehensive review of the literature on socio‐demographic, lifestyle and health determinants associated with MetS and/or frailty [22, 23, 24]. These included age, sex, marital status, fruit and vegetable consumption, alcohol intake, depressive symptoms, quality of life (physical and mental components) and the presence of severe chronic diseases.

Fruit and vegetable consumption was self‐reported with a frequency questionnaire and used as a proxy to capture micronutrient and antioxidant intake, which has been consistently independently associated with prefrailty and frailty risk [25].

Alcohol intake was categorized as none (no alcohol in the previous week), moderate (1–14 units/week) or high (> 14 units/week). Fruit and vegetable consumption was classified as low (< 1 portion/day) or adequate (≥ 1 portion/day).

Quality of life was self‐reported assessed using the SF‐36 physical and mental component scores, categorized into tertiles (physical: < 47.6, 47.7–53.3, > 53.3; mental: < 54.0, 54.1–58.3, > 58.3) [26]. Depressive symptoms were assessed using the CES‐D scale, with scores ≥ 20 indicating an elevated risk of depression, as previously validated [27].

Severe chronic disease was defined as the presence of at least one of the following: coronary artery disease, dementia, stroke, Parkinson's disease or chronic obstructive pulmonary disease (COPD). Participants could present with more than one condition.

### 2.5 Statistical Analyses

Analyses were performed using R software (Version 4.4.3, https://www.r‐project.org/), with the MSM packages for multi‐state modelling [28]. Baseline frailty status (robust, PF‐1, PF‐2, frailty) was described as proportions of study population. Continuous and categorical variables were summarized as means ± standard deviations (SDs) and counts (percentages), respectively. Group differences between participants with and without MetS were assessed using Student's *t*‐tests for continuous variables and chi‐squared tests for categorical variables.

Missing data on covariates were minimal, with no variable exceeding 1.3% missingness. To address these, multiple imputation by chained equations (MICE) with fully conditional specification was applied to generate 10 completed datasets. Because the MSM package does not support Rubin's rules, multi‐state analyses were conducted on a single fully imputed dataset. Although this approach does not fully propagate imputation‐related uncertainty, it preserves all observations and realistic covariate variability while avoiding the limitations of complete‐case analysis (loss of power and bias when data are not missing completely at random [MCAR]) [29] or mean‐value imputation (underestimated variance).

#### 2.5.1 Multi‐State Markov Model

Frailty trajectories were analysed using a continuous‐time, time‐homogeneous Markov multi‐state model, implemented with the MSM package in R [28]. This modelling framework is particularly suitable for panel data, such as in this study, where health states are observed intermittently at fixed examination phases and at subject‐specific time points. The Markov assumption states that future transitions depend solely on an individual's current state and not on the prior sequence of states. This assumption is standard in continuous‐time multi‐state modelling of aging‐related processes, where the current state is assumed to summarize recent health dynamics. However, we acknowledge that the Markov assumption may not fully capture potential history‐dependent effects, such as the influence of time already spent in a given frailty state. This limitation is inherent to continuous‐time multi‐state models with intermittently observed states, where intermediate transitions remain unobserved. Because follow‐up visits were separated by intervals of time, transitions between frailty states could occur between observation points and thus remain unobserved; their probabilities were inferred from the estimated transition intensities.

In this study, five states were defined: robust (State 1), pre‐frail 1 (State 2), pre‐frail 2 (State 3), frail (State 4) and death (State 5). States 1–4 were considered transient, whereas death was defined as an absorbing state. Individuals could transition between adjacent states or from any state directly to death. Once the absorbing state was reached, no further transitions were possible. The restriction to adjacent transitions was defined a priori based on known clinical progression of the frailty phenotype, which typically evolves sequentially as a continuum rather than through abrupt changes between non‐adjacent states [30].

The multi‐state Markov analysis was conducted in two steps. In the first one, a model without covariates was used to estimate transition intensities, transition probabilities over 4‐ and 7‐year periods (corresponding approximately to the first and second follow‐up visits after inclusion for most participants) and the mean sojourn time in each state. This provided a descriptive overview of the overall frailty dynamics. In the second step, covariates were incorporated into the model to estimate the transition‐specific hazard ratios (HRs) with 95% confidence intervals, quantifying the independent effect of exposures on transition risks. All statistical tests were two‐sided, and a *p*‐value < 0.05 was considered statistically significant.

#### 2.5.2 Transition Intensities and Probability Estimation

Transitions between frailty states were modelled using a transition intensity matrix (*Q*), presented below:

$$\left(\begin{array}{cccc} -\left(q12+q15\right) & q12 & 0 & \;0\;q15\\ q21 & -\left(q21+q23+q25\right) & q23 & \;0\;q25\\ 0 & q32 & -\left(q32+q34+q35\right) & \;q34\;q35\\ \begin{array}{c} 0\\ 0 \end{array} & \begin{array}{c} 0\\ 0 \end{array} & \begin{array}{c} q43\\ 0 \end{array} & \begin{array}{c} -\left(q43+q45\right)\;q45\\ \;0\;0 \end{array} \end{array}\right)$$

Each element *q*_*rs*_ represents the instantaneous transition rate from state *r* to state *s*. Let *x*(*t*) = *r* denote the state *r* occupied at time *t*. A transition may occur within the interval (*t*, *t* + Δ*t*), during which an individual can move to another state *s* (*s* = 1 to 5: robust, PF‐1, PF‐2, frail or death).

The *Q* matrix is 5 × 5, with each row summing to zero. Diagonal elements are defined as *q*_*rr*_ = −∑_*s ≠ r*_ *q*_*rs*_. State progression was modelled as a gradual process, consistent with the biological and clinical continuum of frailty. Instantaneous transitions between non‐adjacent states (e.g., robust to PF‐2) were considered implausible and fixed to zero. This assumption aligns with geriatric evidence indicating that frailty progression and recovery generally follow a sequential path [30]. Consequently, the transition intensities *q*_*13*_, *q*_*31*_, *q*_*24*_ and *q*_*42*_ were set to 0. As death is an absorbing state, all transitions from State 5 were also fixed to 0. Model fitting involved estimating the 10 remaining transition intensities (*q*_*12*_, *q*_*15*_, *q*_*21*_, *q*_*23*_, *q*_*25*_, *q*_*32*_, *q*_*34*_, *q*_*35*_, *q*_*43*_ and *q*_*45*_), using maximum likelihood estimation with the quasi‐Newton optimization method. Model adequacy was assessed by verifying model convergence, inspecting the plausibility and stability of estimated transition intensities and comparing observed and model‐predicted transition patterns. In addition, log‐likelihood values were compared across nested models, and the model with the best likelihood and stable convergence properties was retained.

The conditional probability of moving from state *r* to *s*, given that a transition out of state *r* occurs, is defined as *q*_*rs*_ */ (−q*_*rr*_ *)*. This quantity reflects the relative likelihood of moving to state *s* among all possible exit states from *r*. In contrast, absolute transition probabilities over a given time horizon *t* are obtained from the exponential of the intensity matrix, *P*(*t*) = *e* ^Qt^, which accounts for the possibilities of remaining in the same state or transitioning through multiple states during the interval.

Mean residence times correspond to the expected duration spent in a given state before transitioning elsewhere. Under the Markov and time‐homogeneity assumptions, where the transition intensities *qrs* are independent of time *t*, the residence time in each state *r* follows an exponential distribution with mean 1*/(−* $\hat{q}$_*rr*_ *)*, where $\hat{q}$_*rr*_ is the estimated diagonal element of the transition intensity matrix.

#### 2.5.3 Adjustment for Covariates in Transition Intensity Modelling

In the second step of the multi‐state analysis, the effects of covariates on transition intensities were estimated by incorporating clinically relevant variables, together with explanatory variables showing at least a moderate univariate association with frailty transitions (*p* < 0.20). Although MetS or components enter the model as a covariate, they represent the primary exposure of interest in the present analysis. Incorporating time‐varying MetS or components into a continuous‐time multi‐state model would have required substantial restructuring of the data (including interval splitting), which is known to generate a large number of artificial intervals and to produce unstable estimates when the number of observed transitions is limited. In the present study, this issue was particularly relevant for transitions involving frailty states, for which the number of observed transitions was low.

To assess whether this methodological constraint could materially affect our findings, we examined the empirical stability of MetS status and its key components over follow‐up. Among participants with repeated measurements, 73.3% maintained the same MetS status throughout follow‐up, 26.7% experienced at least one change, and true oscillations were uncommon (5.2%). A similar pattern was observed for key MetS components, with abdominal obesity remaining stable in 78.6% of participants (5.2% oscillations) and glycemic status in 72.3% (8.3% oscillations). These exploratory results indicate that MetS status and its key components were largely stable over time, supporting their treatment as baseline exposure specification in the multi‐state model (see Tables S1–S3).

MetS and its components were therefore treated as baseline exposures. All other variables were included as standard baseline covariates for confounding control. The covariates included age, sex, marital status, alcohol intake, fruit and vegetable consumption, depressive symptoms, the physical and mental component scores of the SF‐36 and the presence of severe chronic conditions, as already described. Adjustment for these factors enabled the estimation of the independent effect of each exposure on specific transition risks while controlling for potential confounding. Results are presented as HRs derived from transition intensities, representing the relative instantaneous risk of moving from one frailty state to another according to covariate levels.

To minimize the risk of overfitting and unstable estimates, we applied the commonly recommended rule of at least 8–10 events per variable (EPV) in regression models (logistic or Cox) [31, 32]. When the frequency of observed transition for a given type (e.g., PF‐1 → PF‐2) was limited, the set of covariates was reduced, prioritizing age and sex, followed by clinically relevant factors. This approach ensured model robustness, reduced estimation variance and prevented overparameterization.

#### 2.5.4 Sensitivity Analysis

To assess the robustness of our findings, two sensitivity analyses were performed. First, because some non‐adjacent transitions (e.g., robust → PF‐2 or frailty) were observed in the data, we examined whether allowing such transitions improved model fit. Although these transitions are clinically plausible, they may also reflect unobserved intermediate changes occurring between examination phases. Therefore, the main model restricted transitions to adjacent states. An extended model allowing non‐adjacent transitions was fitted and compared with the base model using a likelihood ratio test (LRT). The result was not statistically significant (*p*‐value = 0.09), indicating no improvement in model fit and supporting the plausibility of the adjacent‐transition structure. Second, to address uncertainty related to missing data, analyses were replicated on two additional imputed datasets generated using the MICE procedure. Because the MSM package does not implement Rubin's rules, the main analysis was based on one fully imputed dataset; replication using alternative imputations yielded qualitatively unchanged results, as expected given the low overall missingness, thereby confirming the robustness of the findings.

## 3 Results

### 3.1 Baseline Characteristics of Enrolled Individuals

A total of 4750 individuals were included in the analysis; 73.9% were male, the median age was 64.6 years, and the mean follow‐up time was 6.9 years. At baseline, 36.9% of participants were MetS. The distribution of pre‐frailty states was as follows: 58.8% robust, 29.4% pre‐frail 1, 9.2% pre‐frail 2 and 2.5% frail. Table S4 summarizes state distributions across follow‐up waves.

Baseline demographic and clinical characteristics according to MetS status are presented in Table 1. Compared with participants without MetS, those with MetS were older (66.9 ± 7.2 vs. 64.8 ± 6.9 years; *p* = 0.0001), more often male (76.2% vs. 72.6%; *p* = 0.008) and less frequently robust (54.2% vs. 61.9%; *p* < 0.0001). They also exhibited a higher prevalence of high alcohol intake (29.3% vs. 25.4%; *p* < 0.0001), former smoking (50.5% vs. 43.4%; *p* < 0.0001) and low fruit and vegetable consumption (< 1 portion/day: 22.6% vs. 20.1%; *p* = 0.046). Mean BMI was higher among participants with MetS (28.4 ± 4.7 vs. 25.4 ± 3.9 kg/m^2^; *p* < 0.0001), as were the prevalences of diabetes (17.2% vs. 1.4%; *p* < 0.0001) and other severe chronic conditions (5.7% vs. 2.9%; *p* < 0.0001). All individual MetS components were more prevalent in the MetS group (*p* < 0.0001). Additionally, both physical and mental health‐related quality of life scores were significantly lower among participants with MetS (both *p* < 0.0001).

**TABLE 1:** Baseline demographic and clinical characteristics of enrolled individuals (*n* = 4750).

Characteristics	Metabolic syndrome		
	No	Yes	*p* ^a^
Age (year, SD)	64.8 (5.7)	66.9 (5.9)	< 0.0001*
Sex (%)			
Men	72.6	76.2	0.008*
Women	27.4	23.8	
Marital status (%)			
Single	11.4	10.5	0.164
Married/cohabitant	77.6	76.8	
Widow/divorced	11.0	12.7	
Frailty states (%)			
Robust	61.9	54.1	< 0.0001*
Pre‐frail 1	28.3	31.6	
Pre‐frail 2	7.8	11.2	
Frail	2.0	3.1	
Alcohol (%)			
No alcohol in the previous week	16.1	18.6	< 0.0001*
Moderate, 1–14 units/week	58.4	52.1	
High, > 14 units/week	25.4	29.3	
Chronic diseases (%)			
No	97.1	94.3	< 0.0001*
Yes	2.9	5.7	
MetS component (%)			
Hyperglycaemia	12.0	45.5	< 0.0001*
Hypertension	38.0	87.5	< 0.0001*
Abdominal obesity	21.1	55.5	< 0.0001*
Hypertriglyceridemia	16.9	91.7	< 0.0001*
Low HDL‐cholesterol	8.9	82.9	< 0.0001*
MetS criteria (%)			
< 3 criteria	100.0	NA	< 0.0001*
3 criteria	NA	50.7	
4 criteria	NA	35.7	
5 criteria	NA	13.7	
BMI (kg/m^2^, SD)	25.4 (3.8)	28.4 (4.3)	< 0.0001*
BMI categories (%)			
≤ 19.9	4.7	1.0	< 0.0001*
20–24.9	45.2	19.9	
25–29.9	39.9	48.2	
≥ 30	10.1	30.8	
Smoking status (%)			
Non‐smoker	51.5	44.6	< 0.0001*
Former smoker	43.4	50.5	
Current smoker	5.1	4.9	
Physical activity level (%)			
Recommendation	59.2	55.2	0.008*
Less than recommendation	40.8	44.8	
Fruit and vegetable intake (%)			
At least once daily	79.9	77.4	0.046*
Less than one daily	20.1	22.6	
Diabetes (%)			
No	98.6	82.8	< 0.0001*
Yes	1.4	17.2	
SF36 physical component score^b^ (%)			
Low	28.0	42.5	< 0.0001*
Medium	33.0	33.8	
High	38.9	23.8	
SF36 mental component score^b^ (%)			
Low	34.1	32.0	< 0.0001*
Medium	34.5	31.4	
High	31.4	36.7	
CESD score^b^			
Non‐case (%)	93.5	92.9	0.424
Case (%)	6.5	7.1	
GHQ score (%)			
No symptom	61.3	58.4	0.0501
At least one symptom	38.7	41.6	

*Note:* Values are sample size (percentages) or mean (SD).

Abbreviation: NA: non‐applicable.

^a^ Chi^2^ (*χ* ^2^) test or Student *t*‐test where appropriate.

^b^ Cut‐off: SF36 physical component score (high, medium, low, this is the cut‐off in tertiles [48.3, 53.8] of the raw score); SF36 mental component score (high, medium, low, this is the cut‐off in tertiles [53.5, 57.9] of the raw score); self‐assessment of depression (< 20, ≥ 20, evaluated by the CESD, the threshold of 20 was used to detect people at risk of depression).

* Significative test.

### 3.2 Transitions Between Frailty States

At the final follow‐up, 45.0% of participants were classified as robust, 32.8% as pre‐frail 1, 13.3% as pre‐frail 2, 4.7% as frail and 4.2% died. Table 2 summarizes the observed transitions between states across the entire follow‐up period, providing a descriptive overview of the cohort's dynamics. Among robust individuals, 24.3% progressed to pre‐frail 1. Of those classified as pre‐frail 1, 34.1% recovered to robustness, whereas 15.4% progressed to pre‐frail 2. Among participants in the pre‐frail 2 state, 32.3% improved to pre‐frail 1, and 11.5% progressed to frailty.

**TABLE 2:** Total observed transitions between frailty states across successive visits.

		Post‐transition, *n* (%)				
		Robust	Pre‐frail 1	Pre‐frail 2	Frail	Death
Pre‐transition, *n* (%)	Robust	3223 (67.0)	1166 (24.3)	248 (5.2)	44 (0.9)	127 (2.6)
	Pre‐frail 1	838 (34.1)	1049 (42.6)	378 (15.4)	109 (4.4)	86 (3.5)
	Pre‐frail 2	107 (14.4)	241 (32.3)	251 (33.7)	86 (11.5)	60 (8.1)
	Frail	6 (3.0)	36 (18.1)	52 (26.1)	69 (34.7)	36 (18.1)

Estimated transition intensities derived from the multi‐state Markov model, accounting for visit timing and unobserved intermediate transitions, are presented in Table 3. Recovery intensities generally exceeded progression intensities. For example, pre‐frail 1 individuals were more likely to recover (0.202; 95% CI: 0.187–0.220) than to progress to pre‐frail 2 (0.156; 95% CI: 0.140–0.173), yielding a recovery‐to‐progression ratio of approximately 1.3. Similarly, the intensity of improvement from pre‐frail 2 to pre‐frail 1 (0.305; 95% CI: 0.267–0.347) was about twice that of progression to frailty (0.151; 95% CI: 0.124–0.184).

**TABLE 3:** Transition intensity between different pre‐frailty states.

Categories transitions					Transition intensity (CI 95%)
Deterioration transitions					
Robust	Pre‐fail 1				0.143 (0.134–0.153)
	Pre‐fail 1	Pre‐fail 2			0.156 (0.140–0.173)
		Pre‐fail 2	Frail		0.151 (0.124–0.184)
Death transitions					
			Frail	Death	0.098 (0.07–0.144)
		Pre‐fail 2		Death	0.149 (0.004–0.05)
	Pre‐fail 1			Death	0.005 (0.001–0.015)
Robust				Death	0.006 (0.004–0.008)
Recovery transitions					
Frail	Pre‐fail 2				0.261 (0.202–0.337)
	Pre‐fail 2	Pre‐fail 1			0.305 (0.267–0.347)
		Pre‐fail 1	Robust		0.202 (0.187–0.220)

*Note:* Red represents transitions corresponding to deterioration or progression to a worse frailty state, whereas green represents recovery transitions to a better frailty state.

### 3.3 Probability of Transitions Between Frailty States

**FIGURE 2:**
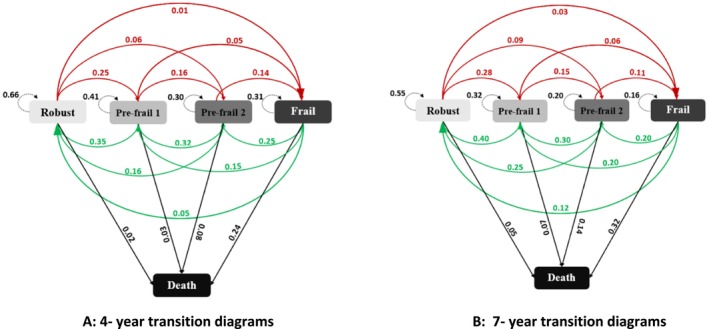
Transition diagrams of frailty states within 4‐year (A) and 7‐year (B) observation intervals. To be specific, in (A), for individuals in pre‐frailty 1 states, the estimated probability of observing maintaining the current state (dashed line from the box of pre‐frailty 1 states), recovery to an robust state (green line from the box of pre‐frailty 1 states) and progression to pre‐frailty 2 states (red line from the box of pre‐frailty 1 states), or death (black lines line from the box of pre‐frailty 1 states) within 4 year was 0.41, 0.35, 0.16 and 0.03, respectively.

Transition probabilities over 4‐ and 7‐year intervals are illustrated in Figure 2. As follow‐up period lengthened, the probabilities of remaining in the same state decreased, whereas adjacent transitions became more frequent. Among robust individuals, the probability of remaining robust over 4 years was 66%, with 25% transitioning to PF‐1; at 7 years, these figures were 55% and 28%, respectively. For PF‐1 participants, 4‐year probabilities were 41% stability, 35% recovery to robustness and 16% progression to PF‐2. At 7 years, 32% stability, 40% recovery and 15% progression. For PF‐2, 4‐year probabilities were 30% remaining in state, 32% reverting to PF‐1 and a 14% deteriorating to frailty; at 7 years, 20% stability, 30% regression and 11% progression. Among frail individuals, 4‐year probabilities were 31% remaining frail, 25% improving to PF‐2 and 24% transitioning to death. At 7 years, these probabilities were 16%, 19% and 32%, respectively. Notably, transitions from frailty to death were the most frequent mortality‐related transitions, increasing from 24% at 4 years to 32% at 7 years. Overall, recovery probabilities were consistently lowest when starting from frailty, compared with those originating from pre‐frailty states.

### 3.4 Effects of MetS and Its Components on Frailty Transitions

Table 4 presents transition‐specific HRs for the associations between MetS and its individual components with transitions between frailty states, adjusted for covariates (full models presented in Tables S5–S10). MetS as a whole was not significantly associated with any specific transition. However, in exploratory analyses examining MetS severity using an ordinal score (0 = 0–2 criteria, 1 = 3 criteria, 2 = 4 criteria, 3 = 5 criteria), the highest severity category (score = 3) was associated with a reduced probability of recovery from frailty to PF‐2 (HR = 0.28; 95% CI: 0.10–0.78; *p* = 0.001); full results are provided in Table S11. Regarding MetS component, abdominal obesity was associated with both greater progression and lower recovery across the frailty states. Specifically, abdominal obesity increased the hazard of transitioning from robust to PF‐1 by 27% (HR = 1.27; 95% CI: 1.09–1.48; *p* = 0.001), reduced the hazard of recovery from PF‐2 to PF‐1 by 28% (HR = 0.72; 95% CI: 0.52–0.99; *p* = 0.048) and halved the likelihood of improvement from frailty to PF‐2 (HR = 0.50; 95% CI: 0.29–0.87; *p* = 0.014). Similarly, hyperglycemia was also associated with a 28% lower hazard of recovery from PF‐1 to robust (HR = 0.72; 95% CI: 0.59–0.88; *p* = 0.001). In contrast, no significant associations were observed for elevated blood pressure, high triglycerides or low HDL cholesterol with any transition. Overall, these results suggest that certain metabolic risk factors, in particularly abdominal obesity and hyperglycemia, may play a dual role, both promoting frailty progression and impeding recovery within the frailty spectrum.

**TABLE 4:** Effects of MetS and components on transitions among frailty states transitions.

Hazard ratio (CI at 95%)											
Exposure		Deterioration transition			Death transition				Recovery transition		
		Robust → pre‐frail 1	Pre‐frail 1 → pre frail 2	Pre‐frail 2 → frail	Robust → death	Pre‐frail 1 → death	Pre‐frail 2 → death	Frail → death	Frail → pre‐frail 2	Pre‐frail 2 → pre‐frail 1	Pre‐frail 1 → robust
MetS	No	Reference			Reference				Reference		
	Yes	1.03 (0.89, 1.2)	1.03 (0.81, 1.3)	1.31 (0.89, 1.9)	1.37 (0.80, 2.3)	1.30 (0.37, 4.6)	0.89 (0.30, 2.7)	1.02 (0.57, 1.8)	0.69 (0.40, 1.2)	0.93 (0.68, 1.3)	0.83 (0.70, 1.0)
Obesity	No	Reference			Reference				Reference		
	Yes	1.27 (1.09, 1.48) *	1.11 (0.8, 1.43)	1.02 (0.69, 1.51)	0.72 (0.3, 1.38)	1.53 (0.49, 4.72)	1.52 (0.43, 5.35)	0.87 (0.51, 1.49)	0.50 (0.29, 0.87) *	0.72 (0.52, 0.99) *	0.90 (0.75, 1.08)
HTA	No	Reference			Reference				Reference		
	Yes	1.07 (0.92, 1.2)	1.13 (0.89, 1.4)	1.01 (0.66, 1.5)	0.95 (0.54, 1.7)	5.86 (0.17, 202.1)	2.42 (0.79, 7.4)	0.84 (0.48, 1.5)	0.69 (0.39, 1.2)	0.96 (0.71, 1.3)	1.01 (0.85, 1.2)
TG	No	Reference			Reference				Reference		
	Yes	1.05 (0.91, 1.2)	1.97 (0.77, 1.2)	1.01 (0.68, 1.5)	1.75 (1.02, 3.0)*	1.27 (0.38, 4.2)	0.57 (0.16, 2.1)	1.15 (0.63, 2.1)	0.71 (0.40, 1.2)	0.92 (0.68, 1.2)	0.95 (0.80, 1.1)
HDL‐chol	No	Reference			Reference				Reference		
	Yes	1.10 (0.95, 1.28)	0.97 (0.77, 1.2)	1.27 (0.85, 1.9)	1.62 (0.95, 2.76)	1.09 (0.34, 3.4)	0.44 (0.07, 2.6)	1.34 (0.72, 2.5)	0.77 (0.44, 1.4)	0.96 (0.71, 1.3)	1.01 (0.84, 1.2)
Glycemia	No	Reference			Reference				Reference		
	Yes	0.93 (0.79, 1.09)	0.96 (0.74, 1.24)	1.08 (0.71, 1.64)	0.63 (0.29, 1.33)	1.71 (0.50, 5.86)	1.23 (0.47, 3.22)	0.66 (0.35, 1.26)	0.90 (0.50, 1.63)	0.86 (0.61, 1.20)	0.72 (0.59, 0.88) *

*Note:* All exposure was adjusted for age, sex, marital status, fruit and vegetable consumption, alcohol intake, depressive symptoms, quality of life (physical and mental components of SF36 questionnaire score) and the presence of severe chronic diseases. Red represents transitions corresponding to deterioration or progression to a worse frailty state, whereas green represents recovery transitions to a better frailty state.

Abbreviation: CI = confidence interval.

* *p* < 0.05.

## 4 Discussion

This longitudinal study, based on a continuous‐time multi‐state Markov model, confirms the bidirectional and potentially reversible nature of frailty states in a British cohort of older adults. It provides refined estimates of transition intensities and probabilities, showing that a substantial proportion of individuals classified as pre‐frail transitioned back to a more robust state over time. Notably, among individuals with one or two frailty criteria, most remained stable, and recovery was more common than progression. In contrast, recovery from frailty was far less frequent, and this state showed the highest probability transition to death, which further increased with longer follow‐up durations.

These findings are consistent with those of Gotaro et al. [17], who, in a meta‐analysis of 16 studies including 42 775 older adults, reported that among pre‐frail individuals, 58.2% remained pre‐frail, 23.1% reverted to robustness and 18.2% progressed to frailty. Together, these results highlight that the transition toward recovery generally exceeded those toward deterioration, supporting the dynamic and reversible nature of early frailty as a modifiable condition and emphasizing its importance as a target for preventive interventions.

In this study, MetS as a whole was not significantly associated with transitions between frailty states. This contrasts with our initial hypothesis, which was based on prior studies reporting MetS as a risk factor for both pre‐frailty and frailty [9, 14]. Several explanations may account for this null association. First, MetS is a heterogeneous syndrome comprising different components (abdominal obesity, dyslipidaemia, hyperglycaemia, hypertension), each potentially exerting different or even opposing effects on frailty transitions. For example, although abdominal obesity has been consistently associated with increased frailty risk [33], other components such as hypertension or elevated triglyceride levels have shown inconsistent associations [14, 34, 35]. Aggregating such divergent risk factors into a single binary classification may obscure true associations by diluting or cancelling out individual effects. Second, substantial variability may exist within each MetS component, depending on treatment status. In the present cohort, a large proportion of participants meeting the dyslipidaemia or hypertension criteria were under pharmacological control, displaying normal blood values, whereas most individuals meeting the hyperglycaemia criterion were untreated (results not shown). Grouping treated and untreated individuals under a single diagnostic label may attenuate associations with frailty outcomes, as it merges heterogeneous phenotypes differing in metabolic profile and degree of clinical control. Consistent with this interpretation, exploratory analyses using an ordinal MetS severity score suggested that only the highest metabolic burden (five criteria) may hinder recovery from frailty, indicating that dichotomizing MetS as a whole may attenuate associations. Taken together, these considerations suggest that a binary MetS variable may inadequately capture the heterogeneity in metabolic burden relevant to frailty dynamics. Future studies should therefore consider individual MetS components and treatment status when examining the metabolic determinants of frailty progression and recovery.

When examining individual MetS components, abdominal obesity emerged as a key determinant of frailty trajectories. It was associated with both increased progression toward more severe states and a reduced hazard of recovery. These findings are consistent with robust evidence linking abdominal obesity to frailty and its core criteria [9, 14, 15], most likely through mechanisms involving insulin resistance [12] and chronic low‐grade inflammation [12, 34]. Several pathological pathways may explain this dual effect. Insulin resistance, frequently present in abdominal obesity, reflects impaired skeletal‐muscle metabolism [36, 37], which in turn reduces protein synthesis and accelerates muscle degradation [36, 37]. This deterioration undermines energy regulation and physical performance, two core components of the frailty phenotype. In parallel, chronic low‐grade inflammation, driven by adipose tissue–derived cytokines, promotes both insulin resistance and sarcopenia, thereby contributing to age‐related declines in strength and mobility [38]. These converging mechanisms provide a coherent explanation for the dual associations of abdominal obesity on frailty dynamics observed in our study.

Hyperglycaemia also emerged as a negative predictor to recovery. Although the underlying mechanisms remain incompletely elucidated, this finding aligns with prior studies reporting elevated blood glucose as a risk factor of frailty [9, 39, 40] and reduced functional capacity [40]. Oleg et al., in a 4.5‐year longitudinal study, found that hyperglycaemia predicted incident frailty in a dose‐dependent manner, even among non‐diabetic individuals [39]. Similarly, Ekram et al. [9], analysing over 18 000 adults aged ≥ 65 years, showed that chronic hyperglycaemia increased the risk of both pre‐frailty and frailty. As with abdominal obesity, these effects are likely mediated through insulin resistance and low‐grade inflammation, which impair muscular and neuromuscular function, thereby limiting the capacity to recover functional reserve.

By contrast, no significant association was observed for hypertension or dyslipidaemia (low HDL‐cholesterol and high triglycerides) with frailty state transitions. This null finding may appear unexpected given prior literature linking these components with frailty risk [9, 14, 15, 33, 34, 35]. However, in the present cohort, most participants classified with dyslipidaemia were receiving lipid‐lowering medication and had normalized lipid profiles, whereas nearly half of those classified as hypertensive were pharmacologically controlled. This high proportion of treated and stabilized individuals likely reduced variability in exposure and may have attenuated potential associations with frailty dynamics.

This study has several limitations. First, although the overall sample size was substantial, it was insufficient to perform adequately powered stratified analyses. It was therefore not possible to explore associations by sex, despite documented sex‐specific differences in frailty risk and its determinants. Similarly, the distinction between treated and untreated individuals within each MetS criterion could not be fully addressed. The multi‐state model requires approximately 8–10 transition events per estimated parameter to ensure model stability, a threshold not met for several subgroups in this dataset. Although this limitation does not compromise the main results, larger studies are needed to test these subgroup‐specific hypotheses. Second, a limitation of this study is that MetS was defined only at baseline, although MetS status may change over time due to lifestyle modification or initiation of cardiometabolic treatments. However, metabolic status in this aging population showed substantial empirical stability. We nevertheless acknowledge that changes related to lifestyle modification or medication initiation could introduce some degree of exposure misclassification. Any such misclassification is expected to be nondifferential with respect to frailty transitions and therefore to bias associations toward the null, suggesting that our estimates may be conservative. Similarly, the weight‐loss component of the frailty phenotype may capture intentional or medication‐related weight loss, particularly among individuals with MetS, but this misclassification is also likely to be nondifferential with respect to frailty transitions and would therefore attenuate rather than inflate associations. Because weight‐loss status is assessed at baseline, prior to the observation of frailty transitions, and because there is no a priori reason to expect that intentional weight loss would be differentially distributed across future frailty trajectories, this type of misclassification is unlikely to be differential. Third, the Whitehall II cohort comprises British civil servants, a socioeconomically advantaged and highly educated population, limiting generalizability of our findings to broader populations. Nonetheless, this cohort provides valuable insights for public health, particularly given the ‘healthy worker effect’ [41], whereby risk factor prevalence tends to be lower than in the general population. Consequently, the associations observed here may be even more pronounced in more vulnerable populations. In addition, the cohort is predominantly male and does not include the most socioeconomically vulnerable older adults, which may further limit the generalisability of our findings to women and to aging populations with different social or health profiles. This is particularly relevant given that women tend to exhibit higher levels of pre‐frailty and frailty in older age; therefore, they follow different frailty trajectories than the predominantly male Whitehall II cohort [42, 43]. Fourth, the relatively long intervals between follow‐up assessments may have missed short‐term fluctuations in pre‐frailty states. However, the extended observation period and high‐quality repeated measures provide a robust temporal foundation for assessing long‐term transition dynamics. From a methodological standpoint, our analyses relied on the Markov and time‐homogeneity assumptions, i.e., that transition rates depend only on the current state and not on the time already spent in that state. Although these assumptions are widely applied in multi‐state modelling, they could not be formally verified in our data and may not fully reflect the complex dynamics of frailty. We also restricted transitions to adjacent states, but sensitivity analyses allowing non‐adjacent transitions yielded similar results, supporting the robustness of our findings.

Despite these limitations, this study provides novel insights into the bidirectional dynamics of frailty, leveraging a large, long‐running aging cohorts with standardized assessments. Future research should incorporate more frequent follow‐up intervals, integrate biological data (e.g., inflammatory markers, hormonal status, sarcopenia measures) and explicitly test time‐dependency in transition models to refine mechanistic understanding and guide targeted preventive strategies.

## 5 Conclusion

This longitudinal analysis demonstrates that, although MetS as a whole is not associated with frailty transitions, specific components, especially abdominal obesity and hyperglycaemia, are strongly linked to both an increased risk of deterioration and a reduced likelihood of recovery. These findings underscore the importance of early identification and tight management of these metabolic disturbances in older adults, in order to strengthen resilience and slow progression toward more advanced stages of frailty, which are less reversible and strongly associated with increased mortality risk.

## Funding

S.K.N.S. received doctoral funding equally supported by Clermont Auvergne Metropole and the Platform of Metabolism Exploration (PFEM, INRAE).

## Ethics Statement

The protocols for data management, data sharing and data preservation in the Whitehall II study strictly adhere to the guidelines established by the Medical Research Council (MRC). Ethical approval for the research, along with written consent from participants, was secured at every stage of the study. The most recent approval was granted by the Joint University College London/University College London Hospital Committee on the Ethics of Human Research (reference number 85/0938).

## Conflicts of Interest

The authors declare no conflicts of interest.

## Supporting information


**Table S1:** Metabolic syndrome status during follow‐up.
**Table S2:** Obesity status during follow‐up.
**Table S3:** Hyperlycemia status during follow‐up.
**Table S4:** Distribution of frailty states in each wave.
**Table S5:** Effects of metabolic syndrome and covariate on frailty state's transitions.
**Table S6:** Effects of obesity and covariate on frailty states transitions.
**Table S7:** Effects of hypertension and covariates on frailty states transitions.
**Table S8:** Effects of hypertriglyceridemia and covariates on frailty states transitions.
**Table S9:** Effects of hyperglycemia and covariates on frailty states transitions.
**Table S10:** Effects of low HDL‐cholesterol and covariates on frailty states transitions.
**Table S11:** MetS gradient effects on frailty states transitions.

## Data Availability

Whitehall II data are available to bona fide researchers for research purposes. Please refer to the Whitehall II data sharing policy at https://www.ucl.ac.uk/psychiatry/research/mental‐health‐older‐people/whitehall‐ii/data‐sharing.
