# Supplementary material for: Metabolic Syndrome as a Determinant of Bidirectional Transitions Between Frailty States: Evidence From the Whitehall II Study
Source: J Cachexia Sarcopenia Muscle. 2026 Jul 8;17(4):e70341. doi: 10.1002/jcsm.70341 (PMC13343728; doi:10.1002/jcsm.70341)
Supplement: Supplementary file 1 — Table S1: Metabolic syndrome status during follow‐up. Table S2: Obesity status during follow‐up. Table S3: Hyperlycemia status during follow‐up. Table S4: Distribution of frailty states in each wave. Table S5: Effects of metabolic syndrome and covariate on frailty state's transitions. Table S6: Effects of obesity and covariate on frailty states transitions. Table S7: Effects of hypertension and covariates on frailty states transitions. Table S8: Effects of hypertriglyceridemia and covariates on frailty states transitions. Table S9: Effects of hyperglycemia and covariates on frailty states transitions. Table S10: Effects of low HDL‐cholesterol and covariates on frailty states transitions. Table S11: MetS gradient effects on frailty states transitions. [file JCSM-17-e70341-s001.docx]

#

# Appendix

**Supplementary Table 1: Metabolic syndrome status during follow-up**

| Category (Metabolic syndrome) | N | % |
| --- | --- | --- |
| Stable (three measurements) | 2,665 | 59.3% |
| Stable (two measurements) | 628 | 14.0% |
| Unstable – single transition (three measurements) | 825 | 18.4% |
| Unstable – two transitions (three measurements) | 232 | 5.2% |
| Unstable (two measurements) | 144 | 3.2% |
| Total* | **4,494** | **100%** |

* Total participants with at least 2 measurements (including baseline)

**Supplementary Table 2: Obesity status during follow-up**

| Category (obesity) | N | % |
| --- | --- | --- |
| Stable (three measurements) | 3,123 | **68.5%** |
| Stable (two measurements) | 462 | **10.1%** |
| Unstable – single transition (three measurements) | 660 | **14.5%** |
| Unstable – two transitions (three measurements) | 236 | **5.2%** |
| Unstable (two measurements) | 79 | **1.7%** |
| Total* | **4,560** | **100%** |

* Total participants with at least 2 measurements (including baseline)

**Supplementary Table 3: Hyperlycemia status during follow-up**

| Category (glycemia) | N | % |
| --- | --- | --- |
| Stable (three measurements) | 2,658 | **59.0%** |
| Stable (two measurements) | 600 | **13.3%** |
| Unstable – single transition (three measurements) | 708 | **15.7%** |
| Unstable – two transitions (three measurements) | 376 | **8.3%** |
| Unstable (two measurements) | 160 | **3.6%** |
| Total* | **4,502** | **100%** |

* Total participants with at least 2 measurements (including baseline)

**Supplementary Table 4:** Distribution of frailty states in each wave

|  | **Robust** | **Pre-frail 1** | **Pre-frail 2** | **Frail** | **Death** | **Total records** |
| --- | --- | --- | --- | --- | --- | --- |
| **Wave 9, n (%)** | 2804 (59.0) | 1404 (29.6) | 429 (9.0) | 113 (2.4) | 0 (0) | 4750 |
| **Wave 11, n (%)** | 2521 (55.5) | 1286 (28.3) | 442 (9.7) | 135 (3.0) | 155 (3.4) | 4539 |
| **Wave 12 n (%)** | 1653 (45.0) | 1206 (32.8) | 487 (13.3) | 173 (4.7) | 154 (4.2) | 3673 |

**Supplementary Table 5:** Effects of Metabolic syndrome and covariate on frailty state’s transitions

| **HAZARD RATIO (CI at 95%)** | | | | | | | | | | | |
| --- | --- | --- | --- | --- | --- | --- | --- | --- | --- | --- | --- |
| Covariables | | Deterioration transition | | | Death transition | | | | Recovery transition | | |
|  |  | Robust->  pre-frail 1 | Pre-frail1-> pre-frail 2 | Pre-frail 2  -> frail | Robust-> death | Pre-frail 1  -> death | Pre-frail 2  -> death | Frail-> death | Frail->  pre-frail 2 | Pre-frail 2-> pre-frail 1 | Pre-frail 1  -> robust |
| MetS | No | Reference |  |  | Reference |  |  |  | Reference |  |  |
|  | Yes | 1.03  (0.89, 1.2) | 1.03  (0.81, 1.3) | 1.31  (0.89, 1.9) | 1.37  (0.80, 2.3) | 1.30  (0.37, 4.6) | 0.89  (0.30, 2.7) | 1.02  (0.57, 1.8) | 0.69  (0.40, 1.2) | 0.93  (0.68, 1.3) | 0.83  (0.70, 1.0) |
| Age |  | 1.16  (1.08, 1.3)* | 1.23  (1.09, 1.4)* | 1.33  (1.11, 1.6)* | 1.99  (1.46, 2.7) | 8.42  (1.82, 38.9)* | 0.74  (0.40, 1.4) | 1.51  (1.04, 2.2)* | 0.83  (0.64, 1.1) | 0.89  (0.77, 1.0) | 0.82  (0.75, 0.9)* |
| Sex | Men | Reference |  |  | Reference |  |  |  | Reference |  |  |
|  | Women | 1.48  (1.22, 1.79)* | 1.12  (0.84,1.48) | 1.05  (0.69,1.58) | 0.56  (0.23,1.37) | 0.14  (0.005, 3.44) | 2.55  (0.83, 7.87) | 0.66  (0.36, 1.22) | 0.55  (0.31,0.96) | 1.19  (0.84,1.67) | 1.36  (1.09,1.69)* |
| Marital status | Single | Reference |  |  | Reference |  |  |  | Reference |  |  |
|  | Married /  cohabitant | 0.85  (0.67, 1.1) | 0.76  (0.54,1.04) | 0.72  (0.42, 1.3) | / | / | / | / | 0.81  (0.38, 1.7) | 1.07  (0.72, 1.6) | 1.29 (0.98, 1.7) |
|  | Widow / divorced | 0.79  (0.58, 1.1) | 0.90  (0.57, 1.4) | 0.74  (0.39, 1.4) | / | / | / | / | 1.55  (0.21, 1.4) | 1.31  (0.76, 2.3) | 1.08  (0.74, 1.6) |
| One portion of fruit and vegetable  Intake daily | Yes | Reference |  |  | Reference |  |  |  | Reference |  |  |
|  | No | 1.17  (0.98, 1.4) | 1.05  (0.81, 1.4) | / | / | / | / | / | / | 0.81  (0.57,1.1) | 0.89  (0.72, 1.1) |
| Depressive^a^ symptom | No | Reference |  |  | Reference |  |  |  | Reference |  |  |
|  | Yes | 1.10  (0.74, 1.6) | 1.12  (0.69, 1.8) | / | / | / | / | / | / | 0.97  (0.57, 1.7) | 1.09  (0.72, 1.6) |
| Alcohol intake^a^ | None | Reference |  |  | Reference |  |  |  | Reference |  |  |
|  | Moderate | 0.71  (0.57, 0.87) | 0.70  (0.51, 0.95) | 0.99  (0.73,1.36) | / | / | / | / | / | 0.83  (0.57,1.21) | 0.98  (0.76,1.26) |
|  | High | 0.72  (0.58, 0.91) | 0.63  (0.44, 0.90) | 1.44  (0.99, 2.1) | / | / | / | / | / | 0.89  (0.58, 1.3) | 0.94  (0.71, 1.2) |
| Chronic diseases^a^ | No | Reference |  |  | Reference |  |  |  | Reference |  |  |
|  | Yes | 0.96  (0.66, 1.40) | 0.92  (0.54, 1.58) | 1.10  (0.64, 1.90) | / | / | / | / | 0.30  (0.09, 0.99)* | 0.80  (0.42, 1.5) | 0.89  (0.55, 1.43) |
| MCS^a^ |  | 0.83  (0.76, 0.90)* | 0.83  (0.72, 0.95)* | / | / | / | / | / | / | 0.90  (0.77, 1.06) | 1.05  (0.95, 1.17) |
| PCS^a^ |  | 0.82  (0.75, 0.89)* | 0.77  (0.68, 0.86)* | / | / | / | / | / | / | 0.97  (0.84, 1.12) | 0.96  (0.86, 1.06) |

“/”: means that, for this transition, no adjustment was made for the corresponding covariate; ^*^(P < 0.05); CI = Confidence Interval. ^a^Depressive symptoms: self-assessment of depression (<20, ≥20, evaluated by the CESD, the threshold of 20 was used to detect people at risk of depression); Alcohol intake (None= No alcohol the previous week, moderate=1–14 units/week, high > 14 unit/week), MCS (Mental component score of the SF-36 health related quality of life questionnaire), PCS (Physical component score of the SF-36 health related quality of life questionnaire)

**Supplementary Table 6:** Effects of Obesity and covariate on frailty states transitions

| **HAZARD RATIO (CI at 95%)** | | | | | | | | | | | | |
| --- | --- | --- | --- | --- | --- | --- | --- | --- | --- | --- | --- | --- |
| Covariables | | Deterioration transition | | | Death transition | | | | Recovery transition | | |  |
|  |  | Robust->  pre-frail 1 | Pre-frail 1-> pre-frail 2 | Pre-frail 2  -> frail | Robust-> death | Pre-frail 1-> death | Pre-frail 2-> death | Frail-> death | Frail->  pre-frail 2 | Pre-frail 2-> pre-frail 1 | Pre-frail 1--> robust |  |
| Obesity | No | Reference |  |  | Reference |  |  |  | Reference |  |  |  |
|  | Yes | 1.27  (1.09, 1.48)* | 1.11  (0.87, 1.43) | 1.02  (0.69, 1.51) | 0.72  (0.37, 1.38) | 1.53  (0.49, 4.72) | 1.52  (0.43, 5.35) | 0.87  (0.51, 1.49) | 0.50  (0.29, 0.87)* | 0.72  (0.52, 0.99)* | 0.90  (0.75, 1.08) |  |
| Age |  | 1.17  (1.09, 1.26)* | 1.23  (1.10, 1.39)* | 1.37  (1.14, 1.6)* | 1.99  (1.46, 2.59)* | 9.10  (2.31, 35.8)* | 0.73  (0.37, 1.45) | 1.47  (1.03, 2.10)* | 0.80  (0.63, 1.02) | 0.88  (0.76, 1.02) | 0.82  (0.75, 0.90)* |  |
| Sex | Men | Reference |  |  | Reference |  |  |  | Reference |  |  |  |
|  | Women | 1.41  (1.16, 1.7)* | 1.12  (0.83, 1.5) | 1.05 (0.69, 1.60) | 0.66  (0.30, 1.4) | 0.15  (0.007, 2.8) | 2.41  (0.71, 8.1) | 0.69  (0.38, 1.2) | 0.68  (0.38, 1.2) | 1.33  (0.92, 1.9) | 1.39  (1.11, 1.7)* |  |
| Marital status | Single | Reference |  |  | Reference |  |  |  | Reference |  |  |  |
|  | Married /  cohabitant | 0.83  (0.65, 1.1) | 0.71  (0.51, 1.0) | 0.84  (0.51, 1.4) | / | / | / | / | 1.02  (0.52, 2.0) | 1.01  (0.67, 1.5) | 1.27  (0.96, 1.7) |  |
|  | Widow / divorced | 0.75  (0.55, 1.01) | 0.88  (0.54, 1.4) | 0.85  (0.47, 1.51) | / | / | / | / | 0.68  (0.27, 1.7) | 1.30  (0.73, 2.31) | 1.02  (0.70, 1.5) |  |
| One portion of fruit and vegetable  Intake daily | Yes | Reference |  |  | Reference |  |  |  | Reference |  |  |  |
|  | No | 1.16  (0.97, 1.40) | 1.05  (0.8, 1.4) | / | / | / | / | / | / | 0.86  (0.6, 1.21) | 0.90  (0.73, 1.11) |  |
| Depressive symptom^a^ | No | Reference |  |  | Reference |  |  |  | Reference |  |  |  |
|  | Yes | 1.09  (0.74, 1.6) | 1.08  (0.66, 1.8) | / | / | / | / | / | / | 0.88  (0.51, 1.52) | 1.07  (0.71, 1.6) |  |
| Alcohol intake^a^ | None | Reference |  |  | Reference |  |  |  | Reference |  |  |  |
|  | Moderate | 0.72  (0.58, 0.88)* | 0.70  (0.52, 0.96)* | 0.94  (0.69, 1.29) | / | / | / | / | / | 0.86  (0.59, 1.26) | 1.00  (0.77, 1.28) |  |
|  | High | 0.73  (0.58, 0.91)* | 0.63  (0.44, 0.90)* | 1.41  (0.97, 2.04) | / | / | / | / | / | 0.92  (0.60, 1.42) | 0.95  (0.72, 1.27) |  |
| Chronic diseases^a^ | No | Reference |  |  | Reference |  |  |  | Reference |  |  |  |
|  | Yes | 0.99  (0.68, 1.4) | 0.96  (0.55, 1.7) | 1.22  (0.69, 2.2) | / | / | / | / | 0.36  (0.11, 1.2) | 0.85  (0.43, 1.7) | 0.89  (0.55, 1.4) |  |
| MCS^a^ |  | 0.82  (0.75, 0.90)* | 0.81  (0.70, 0.94)* | / | / | / | / | / | / | 0.89  (0.76, 1.05) | 1.05  (0.95, 1.17) |  |
| PCS^a^ |  | 0.84  (0.77, 0.91) | 0.77  (0.68, 0.88)* | / | / | / | / | / | / | 0.97  (0.84, 1.12) | 0.97  (0.88, 1.08) |  |

“/”: means that, for this transition, no adjustment was made for the corresponding covariate; ^*^(P < 0.05); CI = Confidence Interval. ^a^Depressive symptoms: self-assessment of depression (<20, ≥20, evaluated by the CESD, the threshold of 20 was used to detect people at risk of depression); Alcohol intake (None= No alcohol the previous week, moderate=1–14 units/week, high > 14 unit/week); Chronic diseases (coronary artery disease, dementia, stroke, Parkinson’s disease, or chronic obstructive pulmonary disease ); MCS (Mental component score of the SF-36 health related quality of life questionnaire), PCS (Physical component score of the SF-36 health related quality of life questionnaire).

**Supplementary Table 7:** Effects of hypertension and covariates on frailty states transitions

| **HAZARD RATIO (CI at 95%)** | | | | | | | | | | | | |
| --- | --- | --- | --- | --- | --- | --- | --- | --- | --- | --- | --- | --- |
| Covariables | | Deterioration transition | | | Death transition | | | | Recovery transition | | |  |
|  |  | Robust->  pre-frail 1 | Pre-frail 1-> pre-frail 2 | Pre-frail 2  -> frail | Robust-> death | Pre-frail 1-> death | Pre-frail 2-> death | Frail-> death | Frail->  pre-frail 2 | Pre-frail 2-> pre-frail 1 | Pre-frail 1--> robust |  |
| HTA | No | Reference |  |  | Reference |  |  |  | Reference |  |  |  |
|  | Yes | 1.07  (0.92, 1.2) | 1.13  (0.89, 1.4) | 1.01  (0.66, 1.5) | 0.95  (0.54, 1.7) | 5.86  (0.17, 202.1) | 2.42  (0.79, 7.4) | 0.84  (0.48, 1.5) | 0.69  (0.39, 1.2) | 0.96  (0.71, 1.3) | 1.01  (0.85, 1.2) |  |
| Age |  | 1.16  (1.08, 1.25)* | 1.22  (1.09, 1.37)* | 1.33  (1.11, 1.61)* | 2.10  (1.56, 2.82)* | 5.58  (1.08, 28.80)* | 0.64  (0.35, 1.17) | 1.58  (1.08, 2.30) | 0.79  (0.62, 1.01) | 0.89  (0.77, 1.03) | 0.82  (0.75, 0.89)* |  |
| Sex | Men | Reference |  |  | Reference |  |  |  | Reference |  |  |  |
|  | Women | 1.47  (1.22, 1.79)* | 1.11  (0.84, 1.47) | 1.05  (0.70, 1.58) | 0.62  (028, 1.38) | 0.08  (0.003, 2.58) | 2.38  (0.84, 6.73) | 0.69  (0.39, 1.19) | 0.55  (0.32, 1.2) | 1.17  (0.83, 1.66) | 1.37  (1.10, 1.72)* |  |
| Marital status | Single | Reference |  |  | Reference |  |  |  | Reference |  |  |  |
|  | Married /  cohabitant | 0.84  (0.66, 1.1) | 0.75  (0.54, 1.0) | 0.81  (0.49, 1.3) | / | / | / | / | 0.98  (0.49, 1.9) | 1.06  (0.72, 1.6) | 1.27 (0.96, 1.7) |  |
|  | Widow / divorced | 0.77  (0.57, 1.04) | 0.93  (0.59, 1.47) | 0.83  (0.46, 1.50) | / | / | / | / | 0.61  (0.24, 1.53) | 1.37  (0.79, 2.39) | 1.04  (0.71, 1.5) |  |
| One portion of fruit and vegetable  Intake daily | Yes | Reference |  |  | Reference |  |  |  | Reference |  |  |  |
|  | No | 1.17  (0.98, 1.40) | 1.03  (0.80, 1.33) | / | / | / | / | / | / | 0.80  (0.57, 1.1) | 0.89  (0.72, 1.1) |  |
| Depressive symptom^a^ | No | Reference |  |  | Reference |  |  |  | Reference |  |  |  |
|  | Yes | 1.09  (0.74, 1.6) | 1.09  (0.67, 1.79) | / | / | / | / | / | / | 0.93  (0.55, 1.6) | 1.07  (0.71, 1.60) |  |
| Alcohol intake^a^ | None | Reference |  |  | Reference |  |  |  | Reference |  |  |  |
|  | Moderate | 0.72  (0.58, 0.88)* | 0.70  (0.52, 0.94)* | 0.97  (0.71, 1.33) | / | / | / | / | / | 0.81  (0.56, 1.18) | 1.01  (0.77, 1.28) |  |
|  | High | 0.73  (0.58, 0.91)* | 0.63  (0.44, 0.90)* | 1.43  (0.98, 2.07) | / | / | / | / | / | 0.88  (0.57, 1.36) | 0.95  (0.72, 1.25) |  |
| Chronic diseases^a^ | No | Reference |  |  | Reference |  |  |  | Reference |  |  |  |
|  | Yes | 0.97  (0.66, 1.42) | 0.90  (0.52, 1.55) | 1.11  (0.65, 1.91) | / | / | / | / | 0.29  (0.10, 1.93) | 0.78  (0.40, 1.51) | 0.89  (0.55, 1.43) |  |
| MCS^a^ |  | 0.82  (0.75, 0.90)* | 0.81  (0.70, 0.94)* | / | / | / | / | / | / | 0.89  (0.76, 1.05) | 1.05  (0.95, 1.17) |  |
| PCS^a^ |  | 0.82  (0.75, 0.89)* | 0.77  (0.68, 0.88)* | / | / | / | / | / | / | 0.97  (0.84, 1.12) | 0.97  (0.88, 1.08) |  |

“/”: means that, for this transition, no adjustment was made for the corresponding covariate; ^*^(P < 0.05); CI = Confidence Interval. ^a^Depressive symptoms: self-assessment of depression (<20, ≥20, evaluated by the CESD, the threshold of 20 was used to detect people at risk of depression); Alcohol intake (None= No alcohol the previous week, moderate=1–14 units/week, high > 14 unit/week); Chronic diseases (coronary artery disease, dementia, stroke, Parkinson’s disease, or chronic obstructive pulmonary disease; MCS (Mental component score of the SF-36 health related quality of life questionnaire), PCS (Physical component score of the SF-36 health related quality of life questionnaire)

**Supplementary Table 8** Effects of hypertriglyceridemia and covariates on frailty states transitions

| **HAZARD RATIO (CI at 95%)** | | | | | | | | | | | | |
| --- | --- | --- | --- | --- | --- | --- | --- | --- | --- | --- | --- | --- |
| Covariables | | Deterioration transition | | | Death transition | | | | Recovery transition | | |  |
|  |  | Robust->  pre-frail 1 | Pre-frail 1-> pre-frail 2 | Pre-frail 2  -> frail | Robust-> death | Pre-frail 1-> death | Pre-frail 2-> death | Frail-> death | Frail->  pre-frail 2 | Pre-frail 2-> pre-frail 1 | Pre-frail 1--> robust |  |
| TG | No | Reference |  |  | Reference |  |  |  | Reference |  |  |  |
|  | Yes | 1.05  (0.91, 1.21) | 0.97  (0.77, 1.23) | 1.01  (0.68, 1.50) | 1.75  (1.02, 3.01)* | 1.27  (0.38, 4.24) | 0.57  (1.16, 2.12) | 1.15  (0.63, 2.10) | 0.71  (0.40, 1.2) | 0.92  (0.68, 1.23) | 0.95  (0.80, 1.13) |  |
| Age |  | 1.16  (1.08, 1.25)* | 1.23  (1.10, 1.38)* | 1.36  (1.13, 1.64)* | 1.97  (1.45, 2.67) | 8.12  (1.88, 35.06) | 0.77  (0.41, 1.45) | 1.46  (0.99, 2.17) | 0.82  (0.64, 1.05) | 0.88  (0.77, 1.02) | 0.82  (0.75, 0.89)* |  |
| Sex | Men | Reference |  |  | Reference |  |  |  | Reference |  |  |  |
|  | Women | 1.49  (1.23, 1.80)* | 1.12  (0.83, 1.48) | 1.03  (0.68, 1.55) | 0.56  (0.23, 1.37) | 0.14  (0.005, 3.35) | 2.67  (0.84, 8.51) | 0.65  (0.35, 1.17) | 0.55  (0.31, 0.95) | 1.18  (0.83, 1.68) | 1.37  (1.10, 1.71)* |  |
| Marital status | Single | Reference |  |  | Reference |  |  |  | Reference |  |  |  |
|  | Married /  cohabitant | 0.84  (0.66, 1.07) | 0.76  (0.54, 1.06) | 0.74  (0.43, 1.28) | / | / | / | / | 0.84  (0.40, 1.78) | 1.06  (0.72, 1.56) | 1.28  (0.97, 1.70) |  |
|  | Widow / divorced | 0.78  (0.57, 1.07) | 0.93  (0.58, 1.49) | 0.75  (0.40, 1.39) | / | / | / | / | 0.56  (0.22, 1.46) | 1.37  (0.78, 2.40) | 1.06  (0.73, 1.53) |  |
| One portion of fruit and vegetable  Intake daily | Yes | Reference |  |  | Reference |  |  |  | Reference |  |  |  |
|  | No | 1.18  (0.99, 1.40) | 1.05  (0.81, 1.37) | / | / | / | / | / | / | 0.81  (0.57, 1.14) | 0.89  (0.72, 1.10) |  |
| Depressive symptom^a^ | No | Reference |  |  | Reference |  |  |  | Reference |  |  |  |
|  | Yes | 1.09  (0.74, 1.61) | 1.13  (0.68, 1.88) | / | / | / | / | / | / | 0.98  (0.56, 1.70) | 1.08  (0.71, 1.62) |  |
| Alcohol intake^a^ | None | Reference |  |  | Reference |  |  |  | Reference |  |  |  |
|  | Moderate | 0.71  (0.58, 0.87)* | 0.70  (0.51, 0.95)* | 0.99  (0.73, 1.36) | / | / | / | / | / | 0.83  (0.57, 1.21) | 0.98  (0.76, 1.27) |  |
|  | High | 0.73  (0.58, 0.92)* | 0.63  (0.44, 0.90)* | 1.50  (1.03, 2.17)* | / | / | / | / | / | 0.88  (0.57, 1.36) | 0.94  (0.71, 1.25) |  |
| Chronic diseases^a^ | No | Reference |  |  | Reference |  |  |  | Reference |  |  |  |
|  | Yes | 0.96  (0.66, 1.40) | 0.94  (0.54, 1.64) | 1.15  (0.67, 1.98) | / | / | / | / | 0.32  (0.09, 1.03) | 0.83  (0.42, 1.62) | 0.89  (0.55, 1.43) |  |
| MCS^a^ |  | 0.83  (0.76, 0.91)* | 0.82  (0.71, 0.94)* | / | / | / | / | / | / | 0.89  (0.75, 1.05) | 1.05  (0.95, 1.17) |  |
| PCS^a^ |  | 0.82  (0.75, 0.89)* | 0.76  (0.67, 0.86)* | / | / | / | / | / | / | 0.97  (0.84, 1.11) | 0.97  (0.87, 1.07) |  |

“/”: means that, for this transition, no adjustment was made for the corresponding covariate; ^*^(P < 0.05); CI = Confidence Interval. ^a^Depressive symptoms: self-assessment of depression (<20, ≥20, evaluated by the CESD, the threshold of 20 was used to detect people at risk of depression); Alcohol intake (None= No alcohol the previous week, moderate=1–14 units/week, high > 14 unit/week); Chronic diseases (coronary artery disease, dementia, stroke, Parkinson’s disease, or chronic obstructive pulmonary disease; MCS (Mental component score of the SF-36 health related quality of life questionnaire), PCS (Physical component score of the SF-36 health related quality of life questionnaire)

**Supplementary Table 9:** Effects of hyperglycemia and covariates on frailty states transitions

| **HAZARD RATIO (CI at 95%)** | | | | | | | | | | | | |
| --- | --- | --- | --- | --- | --- | --- | --- | --- | --- | --- | --- | --- |
| Covariables | | Deterioration transition | | | Death transition | | | | Recovery transition | | |  |
|  |  | Robust->  pre-frail 1 | Pre-frail 1-> pre-frail 2 | Pre-frail 2  -> frail | Robust-> death | Pre-frail 1-> death | Pre-frail 2-> death | Frail-> death | Frail->  pre-frail 2 | Pre-frail 2-> pre-frail 1 | Pre-frail 1--> robust |  |
| Glycemia | No | Reference |  |  | Reference |  |  |  | Reference |  |  |  |
|  | Yes | 0.93  (0.79, 1.09) | 0.96  (0.74, 1.24) | 1.08  (0.71, 1.64) | 0.63  (0.29, 1.33) | 1.71  (0.50, 5.86) | 1.23  (0.47, 3.22) | 0.66  (0.35, 1.26) | 0.90  (0.50, 1.63) | 0.86  (0.61, 1.20) | 0.72  (0.59, 0.88)* |  |
| Age |  | 1.17  (1.09, 1.26)* | 1.22  (1.09, 1.38)* | 1.36  (1.13, 1.64)* | 2.05  (1.51, 2.78)* | 6.41  (1.82, 22.60)* | 0.75  (0.43, 1.30) | 1.57  (1.07, 2.30)* | 0.79  (0.62, 1.01) | 0.88  (0.77, 1.02) | 0.82  (0.75, 0.89)* |  |
| Sex | Men | Reference |  |  | Reference |  |  |  | Reference |  |  |  |
|  | Women | 1.47  (1.21, 1.77)* | 1.11  (0.83, 1.47) | 1.03  (0.68, 1.54) | 0.63  (0.29, 1.37) | 0.17  (0.01, 2.54) | 2.56  (0.88, 7.44) | 0.60  (0.33, 1.09) | 0.57  (0.33, 0.98) | 1.19  (0.84, 1.68) | 1.34  (1.07, 1.67)* |  |
| Marital status | Single | Reference |  |  | Reference |  |  |  | Reference |  |  |  |
|  | Married /  cohabitant | 0.84  (0.66, 1.07) | 0.75  (0.54, 1.03) | 0.82  (0.50, 1.36) | / | / | / | / | 1.001  (0.50, 1.98) | 1.06  (0.72, 1.56) | 1.26  (0.96, 1.67) |  |
|  | Widow / divorced | 0.78  (0.57, 1.06) | 0.94  (0.58, 1.51) | 0.84  (0.46, 1.51) | / | / | / | / | 0.66  (0.26, 1.68) | 1.40  (0.79, 2.47) | 1.05  (0.72, 1.53) |  |
| One portion of fruit and vegetable  Intake daily | Yes | Reference |  |  | Reference |  |  |  | Reference |  |  |  |
|  | No | 1.18  (0.99, 1.41) | 1.03  (0.80, 1.34) | / | / | / | / | / | / | 0.79  (0.55, 1.12) | 0.89  (0.72, 1.11) |  |
| Depressive symptom^a^ | No | Reference |  |  | Reference |  |  |  | Reference |  |  |  |
|  | Yes | 1.09  (0.73, 1.61) | 1.16  (0.70, 1.90) | / | / | / | / | / | / | 0.96  (0.58, 1.7) | 1.08  (0.71, 1.62) |  |
| Alcohol intake^a^ | None | Reference |  |  | Reference |  |  |  | Reference |  |  |  |
|  | Moderate | 0.71  (0.57, 0.87)* | 0.70  (0.51, 0.95)* | 0.97  (0.71, 1.33) | / | / | / | / | / | 0.84  (0.58, 1.22)  ) | 0.98  (0.75, 1.26) |  |
|  | High | 0.72  (0.57, 0.91)* | 0.64  (0.45, 0.91)* | 1.43  (0.99, 2.08) | / | / | / | / | / | 0.91  (0.59, 1.41) | 0.95  (0.71, 1.27) |  |
| Chronic diseases^a^ | No | Reference |  |  | Reference |  |  |  | Reference |  |  |  |
|  | Yes | 0.99  (0.67, 1.47) | 0.92  (0.54, 1.56) | 1.19  (0.69, 2.04) | / | / | / | / | 0.33  (0.11, 1.02) | 0.79  (0.41, 1.51) | 0.91  (0.56, 1.47) |  |
| MCS^a^ |  | 0.83  (0.76, 0.91)* | 0.84  (0.73, 0.96)* | / | / | / | / | / | / | 0.91  (0.78, 1.07) | 1.05  (0.95, 1.17) |  |
| PCS^a^ |  | 0.82  (0.75, 0.89)* | 0.76  (0.67, 0.86)* | / | / | / | / | / | / | 0.97  (0.84, 1.11) | 0.97  (0.87, 1.07) |  |

“/”: means that, for this transition, no adjustment was made for the corresponding covariate; ^*^(P < 0.05); CI = Confidence Interval. ^a^Depressive symptoms: self-assessment of depression (<20, ≥20, evaluated by the CESD, the threshold of 20 was used to detect people at risk of depression); Alcohol intake (None= No alcohol the previous week, moderate=1–14 units/week, high > 14 unit/week); Chronic diseases (coronary artery disease, dementia, stroke, Parkinson’s disease, or chronic obstructive pulmonary disease; MCS (Mental component score of the SF-36 health related quality of life questionnaire), PCS (Physical component score of the SF-36 health related quality of life questionnaire) .

**Supplementary Table 10:** Effects of Low HDL-cholesterol and covariates on frailty states transitions

| **HAZARD RATIO (CI at 95%)** | | | | | | | | | | | | |
| --- | --- | --- | --- | --- | --- | --- | --- | --- | --- | --- | --- | --- |
| Covariables | | Deterioration transition | | | Death transition | | | | Recovery transition | | |  |
|  |  | Robust->  pre-frail 1 | Pre-frail 1-> pre-frail 2 | Pre-frail 2  -> frail | Robust-> death | Pre-frail 1-> death | Pre-frail 2-> death | Frail-> death | Frail->  pre-frail 2 | Pre-frail 2-> pre-frail 1 | Pre-frail 1--> robust |  |
| HDL-Chol | No | Reference |  |  | Reference |  |  |  | Reference |  |  |  |
|  | Yes | 1.10  (0.95, 1.28) | 0.97  (0.77, 1.23) | 1.27  (0.85, 1.9) | 1.62  (0.95, 2.76) | 1.09  (0.34, 3.43) | 0.44  (0.07, 2.6) | 1.34  (0.72, 2.5) | 0.77  (0.44, 1.4) | 0.96  (0.71, 1.30) | 1.01  (0.84, 1.2) |  |
| Age |  | 1.16  (1.08, 1.25)* | 1.23  (1.10, 1.38)* | 1.32  (1.10, 1.59)* | 1.93  (1.42, 2.63)* | 8.37  (1.98, 35.35)* | 0.78  (0.42, 1.45) | 1.43  (0.99, 2.08) | 0.81  (0.63, 1.03) | 0.89  (0.77, 1.02) | 0.82  (0.75, 0.89)* |  |
| Sex | Men | Reference |  |  | Reference |  |  |  | Reference |  |  |  |
|  | Women | 1.49  (1.23, 1.81)* | 1.13 (0.85, 1.50) | 1.04 (0.68, 1.57) | 0.55  (0.22, 1.37) | 0.16  (0.07, 3.19) | 2.63  (0.89, 7.71) | 0.67  (0.38, 1.19) | 0.55  (0.31, 0.97)* | 1.20  (0.85, 1.70) | 1.38  (1.11, 1.72)* |  |
| Marital status | Single | Reference |  |  | Reference |  |  |  | Reference |  |  |  |
|  | Married /  cohabitant | 0.84  (0.66, 1.08) | 0.75 (0.54, 1.04) | 0.76  (0.45, 1.27) | / | / | / | / | 0.90  (0.43, 1.87) | 1.06  (0.72, 1.56) | 1.29 (0.98, 1.7) |  |
|  | Widow / divorced | 0.78  (0.57, 1.07) | 0.91  (0.58, 1.44) | 0.76  (0.42, 1.40) | / | / | / | / | 0.60  (0.23, 1.53) | 1.33  (0.76, 2.31) | 1.06  (0.73, 1.53) |  |
| One portion of fruit and vegetable  Intake daily | Yes | Reference |  |  | Reference |  |  |  | Reference |  |  |  |
|  | No | 1.18  (0.98, 1.4) | 1.05  (0.81, 1.38) | / | / | / | / | / | / | 0.81  (0.57, 1.14) | 0.89  (0.72, 1.1) |  |
| Depressive^a^ symptom | No | Reference |  |  | Reference |  |  |  | Reference |  |  |  |
|  | Yes | 1.09  (0.74, 1.61) | 1.12  (0.68, 1.84) | / | / | / | / | / | / | 0.97  (0.56, 1.66) | 1.08  (0.71, 1.62) |  |
| Alcohol intake^a^ | None | Reference |  |  | Reference |  |  |  | Reference |  |  |  |
|  | Moderate | 0.71  (0.57, 0.87)* | 0.69  (0.51, 0.95)* | 1.01  (0.74, 1.38) | / | / | / | / | / | 0.83  (0.57, 1.21) | 0.98  (0.76, 1.27) |  |
|  | High | 0.72  (0.57, 0.91)* | 0.63  (0.44, 0.90)* | 1.50  (1.03, 2.17)* | / | / | / | / | / | 0.88  (0.57, 1.36) | 0.94  (0.70, 1.25) |  |
| Chronic diseases^a^ | No | Reference |  |  | Reference |  |  |  | Reference |  |  |  |
|  | Yes | 0.95  (0.65, 1.39) | 0.94  (0.54, 1.62) | 1.07  (0.63, 1.84) | / | / | / | / | 0.30  (0.09, 0.95)* | 0.81  (0.42, 1.59) | 0.88  (0.55, 1.41) |  |
| MCS^a^ |  | 0.83  (0.76, 0.91)* | 0.82  (0.71, 0.94)* | / | / | / | / | / | / | 0.89  (0.76, 1.05) | 1.05  (0.95, 1.17) |  |
| PCS^a^ |  | 0.82  (0.75, 0.89)* | 0.76  (0.68, 0.86)* | / | / | / | / | / | / | 0.97  (0.84, 1.12) | 0.97  (0.88, 1.08) |  |

“/”: means that, for this transition, no adjustment was made for the corresponding covariate; ^*^(P < 0.05); CI = Confidence Interval. ^a^Depressive symptoms: self-assessment of depression (<20, ≥20, evaluated by the CESD, the threshold of 20 was used to detect people at risk of depression); Alcohol intake (None= No alcohol the previous week, moderate=1–14 units/week, high > 14 unit/week); Chronic diseases (coronary artery disease, dementia, stroke, Parkinson’s disease, or chronic obstructive pulmonary disease; MCS (Mental component score of the SF-36 health related quality of life questionnaire), PCS (Physical component score of the SF-36 health related quality of life questionnaire)

**Supplementary Table 11:** MetS gradient effects on frailty states transitions

| **HAZARD RATIO ( CI at 95%)** | | | | | | | | | | | |
| --- | --- | --- | --- | --- | --- | --- | --- | --- | --- | --- | --- |
| Exposure | | Deterioration transition | | | Death transition | | | | Recovery transition | | |
|  |  | Robust->  Pre-frail 1 | Pre-frail 1->  Pre frail 2 | Pre-frail 2-> Frail | Robust-> death | Pre-frail 1-> death | Pre-frail 2-> death | Frail-> death | Frail->  Pre-frail 2 | Pre-frail 2-> Pre-frail 1 | Pre-frail 1-> Robust |
| MetS | No | Reference |  |  | Reference |  |  |  | Reference |  |  |
|  | 3 criteria | 0.96 (0.80, 1.2) | 0.89 (0.66, 1.2) | 1.42 (0.84, 2.4) | 1.53 (0.84, 2.8) | 0.59 (0.06, 5.4) | 0.99 (0.24, 4.0) | 1.21 (0.61, 2.4) | 0.88 (0.41, 1.9) | 0.94 (0.63, 1.4) | 0.88 (0.71, 1.10) |
|  | 4 criteria | 1.11 (0.89, 1.40) | 1.22 (0.87, 1.70) | 1.44 (0.85, 2.50) | 1.10 (0.45, 2.70) | 1.36 (0.33, 5.60) | 0.69 (0.08, 5.90) | 1.01 (0.44, 2.30) | 0.93 (0.44, 2.00) | 0.99 (0.65, 1.50) | 0.82 (0.62, 1.10) |
|  | 5 criteria | 1.10 (0.78, 1.54) | 1.11 (0.69, 1.78) | 1.20 (0.69, 2.09) | 1.53 (0.48, 4.90) | 2.21 (0.43, 11.23) | 1.14 (0.23, 5.68) | 0.69 (0.28, 1.70) | **0.28 (0.10, 0.78)** | 0.82 (0.43, 1.57) | 0.70 (0.45, 1.08) |

Was adjusted for age, sex, marital status, fruit and vegetable consumption, alcohol intake, depressive symptoms, quality of life (physical and mental components of SF36 questionnaire score), and the presence of severe chronic diseases. Bold: P < 0.05; CI = Confidence Interval.
